# Development, implementation, and pilot study of a sentinel network ("The Watchtowers") for monitoring emergency primary health care activity in Norway

**DOI:** 10.1186/1472-6963-8-62

**Published:** 2008-03-26

**Authors:** Elisabeth Holm Hansen, Steinar Hunskaar

**Affiliations:** 1 National Centre for Emergency Primary Health Care, Kalfarveien 31, NO-5018 Bergen, Norway; 2 Section for General Practice, Department of Public Health and Primary Health Care, University of Bergen, Kalfarveien 31, NO-5018 Bergen, Norway

## Abstract

**Background:**

In Norway there is a shortage of valid health activity statistics from the primary care out-of-hours services and the pre-hospital emergency health care system. There is little systematic information available because data registration is lacking or is only recorded periodically, and definitions of variables are not consistent.

**Method:**

A representative sample of Norwegian municipalities and out-of-hours districts was contracted to establish a sentinel network, "The Watchtowers", and procedures were developed for collecting continuous data from out-of-hours services. All contacts, either per telephone or direct attendance, are recorded during day and night. The variables are registered in a computer program developed by the National Centre for Emergency Primary Health Care, and sent by email in Excel-file format to the Centre on a monthly basis.

**Results:**

The selection process yielded a group of 18 municipalities, with a fair degree of representativeness for Norwegian municipalities as a whole. The sample has 212,921 inhabitants, which constitutes 4.6% of the total Norwegian population. During a pilot period lasting three months the Watchtowers recorded all individual contacts. The procedures for registration, submitting and checking data worked satisfactorily. There was little data missing, and during the last three months of 2006 a total of 23,346 contacts were registered.

**Conclusion:**

We have been able to establish a sentinel network with a fair degree of representativeness for Norwegian out-of-hours districts and municipalities. The data collected reflect national activities from casualty clinics in Norway. Such data are useful for both research and system improvements.

## Background

In many countries primary health care activity data are scarce or unreliable due to lack of comprehensive data collection [1,2]. The need for valid empirical data from such services also is obvious for several reasons; national statistics, public health or local organisational planning, for research, health care priorities, and health care policy making. Depending on their existence in a particular country or area, activity data from this part of the health care system may be obtained from several sources such as patient registries, national morbidity and mortality registries, prescription databases, reimbursement claims, continuous and prospective activity data registration and others [2]. In many countries the denominator for rates may be difficult to obtain for studies in primary care, but it is anyhow important to define both for research and for statistical purposes [3].

In Norway, like in most other countries, there is a lack of valid health activity statistics from the primary care out-of-hours services and pre-hospital emergency health care system. There is little systematic information available because data registration is lacking or is only recorded periodically, and definitions of variables may vary. Data from this service is important, however, e.g. for national plans and emergency primary health care functions [3-7].

The Norwegian Ministry of Health and Care Services has established The National Centre for Emergency Primary Health Care [8]. The Centre is academically connected to the Department of Public Health and Primary Health Care at the University of Bergen and to the National Centre on Emergency Health Care Communication. The purpose of the Centre is to establish and disseminate knowledge of emergency primary health care through multi-disciplinary research and information dissemination activities. The Norwegian Directorate for Health and Social Affairs has emphasised the importance of comparison data in the out-of-hours services and that such data should be of relevant content and of good quality.

In lack of comprehensive national data sets usable for obtaining routine valid statistics with a uniform variable set and strict definitions, the Centre has initiated an enterprise called "The Watchtowers" which aims at including a representative sample of Norwegian municipalities. The purpose is to provide routine information over several years, based on a limited or minimal dataset, which will enable the monitoring, evaluation and comparison of the respective activities in the area of primary health care emergency services. This paper describe the organisation, variable set and sampling of the Watchtowers, and also provide results from the pilot study period during the last three months of 2006.

## Methods

### Organisation of emergency services in Norway

Norway has a two-level public health care system, with only a marginal private sector. The four geographically based Regional Health Authorities (RHAs) owned by the Ministry of Health and Care Services maintain the hospital sector, including all ambulance services and the National air ambulance services. The RHAs also organise and run the 20 regional Emergency Medical Communication Centres (EMCC) including maintenance of the emergency call number 113. These services, including university hospitals, form the secondary and tertiary health care system.

The 431 municipalities (2007) are by law in charge of organising primary health care, including general practice, nursing homes, home care, preventive medicine for children (including prenatal care, but not deliveries), school health care, and also local emergency medical services for all inhabitants 24 hours a day. The emergency medical service is usually managed by the General Practitioners' (GPs') surgeries during the office hours and by municipality maintained out-of-hours duties by GPs during evenings, nights and weekends, often based in local casualty clinics [5]. Each municipality also has a duty to maintain one specific telephone number at a local Emergency Medical Communication Centre (LEMCC) (usually located in the casualty clinic) for cases that are urgent but not life threatening. Both the local emergency care system (LEMCC) and the EMCCs are staffed with nurses who use telephone triage to prioritise patient treatment and/or transportation. After assessment of the patient's health problem, a decision is made about the appropriate level of action, which could include advice only, home visit by a GP, appointment with a GP in a casualty clinic, a call out for the GP on duty or an ambulance or urgent transportation to hospital by land, sea or air ambulance service [7].

### Norwegian casualty clinics and GPs

In 2006 there were 262 out-of-hours districts in Norway, constituted either by one municipality alone or by inter-municipality co-operatives [5,6]. Out-of-hours emergency primary health care service is thus inter-municipality based in two thirds of Norwegian municipalities. Regular GPs participate in this service to varying degrees, although mandatory contracted to it [9]. In half of the municipalities all regular GPs take out-of-hours shifts. There are substantial variations in the annual number of phone calls per inhabitant to municipal out-of-hours services [7]. Due to Norwegian geographical factors, there are also large variations in patient transport time and the availability of ambulances [5].

Most out-of-hours services are located in a casualty clinic in the host municipality, but some use GPs' surgeries as location. A closed and nation-wide medical radio network is used for communication between doctors on call, ambulances, LEMCC and the EMCC. When an emergency situation occurs, simultaneous radio alarms will be transmitted to both the GP on call and the ambulances in the actual area. A study from 2005 showed, however, that only half of the doctors on duty were available on the medical radio network all the time, despite it being mandatory to be so [5].

### Sample of municipalities participating in the Watchtower project

Participation in The Watchtower project is based on motivation and voluntary contracts. In 2005 all the 433 municipalities in Norway were invited by the Centre to participate in the project. After three invitations with repeated and a gradually increasing amount of information about the project being distributed, 44 municipalities remained for the selection process and final inclusion. In order to select a sample as representative as possible for Norwegian municipalities as a whole and also reflecting the different organisational models for emergency primary health care, these 44 municipalities were categorised through several statistical dimensions defined and managed by Statistics Norway [10].

The following variables were used in the selection process, here presented with most recent available data:

- Population size in absolute number of inhabitants and also categorised into small, medium and large. Municipalities with < 5,000 inhabitants are defined as small, those with 5,000 to 19,999 are medium, and municipalities with 20,000 or more inhabitants are large

- Change in population (%) between 2001 and 2006 (quartiles)

- Proportion of inhabitants 0–17 years of age and 67 years and over (quartiles)

- Gender distribution (quartiles)

- Degree of centralisation of population in municipality (graded from 1–7)

- Statistics Norway's compound classification for municipalities (graded 1–10)

- Distribution of employment by branches of business and industry (quartiles)

- Municipality's public economy (quartiles)

- Gross income among men (quartiles)

It was anticipated that there should be between 0.5 and 1.0 annual contact per inhabitant to the out-of-hours services. In order to obtain approximately 100,000 cases as a minimum, it was decided to include a total of between 150,000 and 250,000 inhabitants in the project.

The selection process resulted in a specific invitation to a sample consisting of seven casualty clinics with a total of 18 municipalities from different parts of Norway. All agreed to participate and were contracted for participation on a long term basis. Each Watchtower is paid a small amount of money based on number of inhabitants to cover administrative and other running costs. No reimbursement for workload is provided. Total costs for the project is 0.65 EUR per case registered.

### Data collection

A set of ten variables was developed by expert opinion. No clinical data are recorded. For every contact or patient's request for help from the out-of-hours service the following ten variables are recorded:

- Nationality and place of residence (municipality name and number) of the patient

- Time of contact: Year, number of week in the year (x/52), number of day in the week (x/7), and time of the day (daytime 08.00–15.29, afternoon 15.30–22.59, and night 23.00–07.59)

- Gender of patient

- Age of patient, registered as attained years. A child of less than one year is registered with the value zero

- Mode of contact: Telephone contact, direct attendance to the casualty clinic, contact by health professionals, contact by EDCs or others (for example police)

- First response initiated by given categories: Telephone advice by a nurse, telephone advice by a doctor, medical examination by a doctor, medical consultation by a nurse, home visit by a doctor, acute response by ambulance and doctor, and others (e.g. sending ambulance without a doctor, referring to police or to a regular GP on daytime)

- Priority degree according to the Norwegian Index for Medical Emergency Assistance [11].

Norwegian Index for Medical Emergency Assistance is a decision tool to ensure an appropriate response to a medical emergency. The Index is used in all EMCCs and is also available in all casualty clinics in Norway, but is not mandatory in the latter. The Index is intended to regulate or standardise the quality of medical evaluation performed by nurses in the EMMC/LEMCC. Each call to or contact with a Watchtower is classified by priority degree according to the Index with colour codes "Red", Yellow" or "Green. Red colour is defined as an "acute" response, with the highest priority. Yellow colour is defined as an "urgent" response, with a high, but lower priority. Green colour is defined as a "not urgent" response, with the lowest priority.

All contacts, both per telephone or attendance, are recorded during day and night by the attending nurses. The variables are registered in a computer program developed by the Centre, and sent in Excel-file format monthly by email to the Centre. One appointed nurse/co-ordinator at each casualty clinic is responsible for checking and sending data locally. In some of the Watchtowers, checks for completeness of registrations in order to avoid under-reporting can be made because of simultaneous registration on paper sheets or digitally for other purposes. Such checks are not part of the project's procedures, but each Watchtower is encouraged to perform them if possible. Strict procedures are followed for sending, receiving and checking data, including checking for completeness, running frequency tables, identifying missing and duplicate records, and checking for invalid variable values. All data are subsequently merged into a master database.

### Data quality

We did a preliminary four week pilot study in April 2006. The aim of the pilot study was to ensure good quality of the data, evaluate procedures for transmitting data, test the registration workload, and obtain a preliminary indication of the annual number of cases. Three casualty clinics participated in the pilot study, one clinic in a small district, one clinic in a city and one large clinic consisting of seven municipalities [7]. Our conclusion was that the system was working well, that the clinics were able to comply with the procedures, and that it was possible to sustain the project over a long period of time.

During the last three months of 2006 we carried out a new pilot study with all the recently selected seven Watchtowers. The Centre arranged meetings with the respective casualty clinics, their leaders and many of the nurses before commencing the pilot study. In this pilot phase, procedures were tested and some clarifications of the variable definitions were made.

The statistical analyses were performed using Statistical Package for the Social Sciences (SPSS version 13). Standard univariate statistics were used to characterise the sample, including simple distributions and standard cross tables. The Watchtower project is approved by The Norwegian Data Inspectorate.

## Results

The selected seven casualty clinics covering 18 municipalities are located from Alta in north of Norway to Arendal in the south, and they represent different sizes of out-of-hours districts, single and inter-municipality cooperatives, and a wide variety of dimensions included in the selection process (Table 1). The largest Watchtower contains ten municipalities (Arendal), the smallest is a municipality consisting of a group of islands in the western part of Norway (Austevoll, 4,391 inhabitants). The Watchtowers had a total of 212,921 inhabitants per January 1, 2006, which constituted 4.6% of the Norwegian population and covered 4.1% of the total Norwegian area.

**Table 1:** Characteristics of the 7 Watchtowers and the constituting 18 municipalities. Data per 01.01.2006, otherwise stated.

**Watchtower**	**Population**	**Area (km^2^)**	**Municipality**	**Population categories (1–3)**	**Change in population, 2001 to 2006 (quartiles)**	**Gross income, men 17 years + 2004 (quartiles)**	**Centralisation classification (1–7), 1994**
Nes	18,022	637	Nes	2	4	3	7
Solør	20,646	2,583	Grue	2	1	2	4
			Åsnes	2	1	1	4
			Våler	1	2	1	4
Arendal	85,431	5,622	Arendal	3	3	3	6
			Fyresdal	1	3	1	1
			Risør	2	2	2	5
			Grimstad	2	4	4	7
			Nissedal	1	1	2	6
			Gjerstad	1	3	2	4
			Vegårdshei	1	3	2	6
			Tvedestrand	2	2	3	6
			Froland	1	4	3	6
			Åmli	1	1	1	1
Kvam	8,306	616	Kvam	2	2	3	2
Austevoll	4,391	117	Austevoll	1	2	4	2
Tromsø	63,596	2,566	Tromsø	3	4	3	7
Alta	17,889	3,849	Alta	2	4	2	3

Norway	4,640,200	385,199					

### Representativeness of the municipalities

The strategic selection process resulted in a group of municipalities with a fair representativeness for Norwegian municipalities as a whole. All population sizes are represented among the Watchtowers and the distribution is close to the national, although a large city is lacking.

For most of the demographic and socio-economical dimensions the categories are well distributed (all detailed data are not shown in text or table). The four categories for change in population between 2001 and 2006 are represented with 22–28% each. The same is found for proportions of persons 0–17 years of age. For inhabitant 67 years and older our sample of quartiles is somewhat skewed, but compared to the median national distribution, our sample has a mere 6% higher proportion of that age group. We have fewer municipalities in the highest quartile of average gross income among men. All centralisation categories are represented in our sample, but compared to Norway in general our sample have somewhat fewer municipalities with the lowest centralisation code. The number of women per 100 men in Norway is 102, while in our sample it is 99.7. Employment categories in primary, secondary and tertiary levels of trades and industries are well distributed among the participating municipalities and Watchtowers.

### Pilot study: Data collection and missing data

**Figure 1:**
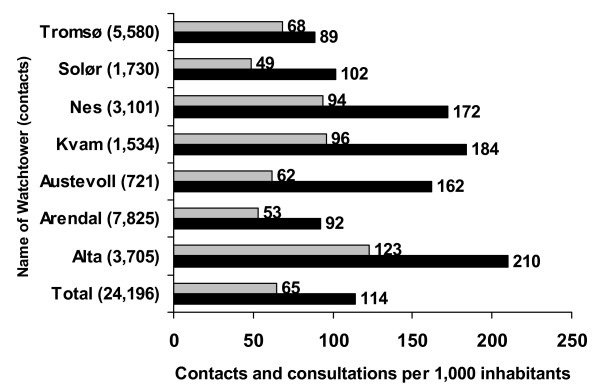
Contacts and consultations per 1,000 inhabitants for the out-of-hours districts the last three months of 2006.

A total of 23,346 contacts were registered during the last 3 months of 2006. The data were submitted to the Centre in accordance with established procedures. Data check revealed no missing data for the variables nationality, municipality name and municipality number. Time of contact, number of week, day and time were also complete. We found 1.5% missing registrations for mode of contact, 1.5% for first response initiated, 1.3% for gender, 2.0% for age, and 2.3% missing for priority degree. We discovered that approximately 850 cases were lost from one casualty clinic (15.2% of cases from that clinic) due to a technical mishap that could not be reversed. The number of missing cases is included when the contact rates and consultations per 1,000 inhabitants are presented. The total number of cases is therefore 24,196 in Figure 1.

### Contacts to the Watchtowers in the pilot study period

There were large differences in total contact rates (89–210) and in clinical consultations (53–123) per 1,000 inhabitants (Figure 1). Daytime contact rates varied from 3 to 72 per 1,000 inhabitants (further data not shown). A total of 67.3% of all contacts were made by telephone and 23.7% by direct attendance to the clinics, while 9.0% of all contacts were made by health professionals and police. Table 2 shows age group and gender for the patients, priority degree, and actions taken for the total sample and the range between the seven Watchtowers. A large majority of the contacts were classified as lowest priority. The rate of home visits per 1,000 inhabitants was low (1 to 3) in all the casualty clinics except for one (16.5). Based on the numbers from the pilot study (disregarding any seasonal variation) it can be estimated that the out-of-hours casualty clinics in Norway have a mean of 0.45 contacts per inhabitant per year, which gives a national estimate for Norway of about 2.1 million contacts per year.

**Table 2:** Distribution and range of age and gender of the patients, priority grade and action taken, for the seven Watchtowers (%).

**Variable**	**Distribution**	**Range between the Watchtowers**
**Age group **(years) (n = 22,873)		
0–17	30.9	20.9–33.2
18–66	54.1	46.4–61.3
67 and over	15.0	9.2–28.2
		
**Gender **(n = 23,032)		
Male	45.0	40.3–48.6
Female	55.0	51.4–59.7
		
**Priority grade **(n = 22,802)		
Acute	2.4	1.6–3.7
Urgent	17.7	10.8–30.8
Not urgent	79.9	65.5–87.6
		
**Actions taken **(n = 22, 994)		
Telephone advice by nurse	19.6	15.3–27.4
Telephone advice by doctor	10.3	4.1–20.7
Appointment with doctor	60.4	38.7–76.8
Consultation by nurse	1.8	1.0–5.1
Call out GP and ambulance	2.1	1.3–4.7
Home visit by doctor	1.9	0.1–16.5
Others	3.9	1.0–9.5

## Discussion

Through the Watchtower project the National Centre for Emergency Primary Health Care has been able to establish and quality check a large and representative monitoring system for providing routine data based on a minimal data set for primary health care emergency services in Norway. This long-term enterprise will enable a monitoring, evaluation and comparison of the respective variables in the area of out-of-hours services, based on information from about 100,000 contacts a year.

Ideally, all out-of-hours districts and casualty clinics should be able to report contacts, and in addition some clinical data, by a common data sheet and by defined variables. This is not currently possible in Norway, nor will it be in the near future. Secondary, one could have established a large randomised sample of municipalities and hoped for their participation and cooperation in the project. We decided not to attempt such a strategy, as we anticipated low response rates, which would entail greater representativeness problems than the strategic selection process that was in fact chosen. The results indicate that our Watchtowers comprise a sample of municipalities and out-of-hours clinics with a satisfactory representativeness relative to the geographical, demographic, and socio-economical variables that were used. There is no knowledge of how contact rates may vary along with such variables nation-wide, so hopefully the size of population chosen together with the representativeness of the variables measured will secure data with adequate generalisation potential. An indirect proof of good match is the fact that on the basis on data from the Watchtowers we can estimate a total of 1.27 million consultations with a GP during out-of-hours services in Norway in 2006. Real data from the Norwegian Labour and Welfare Organisation show that 1.30 million reimbursement claims were submitted for such consultations, an almost identical figure.

We use a rather minimal data set. At an early planning stage of the project it was claimed that it would only be possible to obtain few data that could be registered simultaneously with the incident, e.g. the presentation by the patient through telephone or attendance. Therefore, there are no diagnoses or other clinical data that need to be collected from the patients' files. Reason for encounter might have been included, but this was declined for practical and methodological reasons. Also, if we wanted to include clinical data, we would have had to obtain more ethical data protection approvals. The outcome of such applications is uncertain. All data are recorded anonymously; it is thus impossible to analyse multiple contacts from the same person.

The experiences from the data collection procedures and checks for missing data are satisfactory. The discovery of lost cases shows that the pilot study was useful and that the security checks worked, and we could adjust the procedures. However, we lack validation data for two important variables. First, we have no system for finding or retrieving data due to under-reporting. During busy periods on duty or due to absent-mindedness, some contacts may be missed for registration. The project group will try to establish a validation procedure in order to obtain data on this possible problem. Second, the assessment of priority degree is carried out by a large number of nurses based on criteria that are not distinct. Differences in priority degree between the Watchtowers may therefore be due to both local real differences and differences in thresholds for grading. A study from the Netherlands showed that triage nurses may both underestimate and overestimate the level of emergency [12]. Validation projects and harmonisation efforts with regard to this topic should therefore be initiated within the Watchtower project.

To uphold motivation, avoid mistakes in definitions and avoid missing cases, the Watchtowers are subject to continuous contact with and surveillance from the Centre. Such efforts have been important, according to the literature [13].

Several models for obtaining routine data from primary health care services are reported from other countries. Examples are "Sentinel practice networks", networks of practices or municipalities that monitor one or more specific illness problems on a regular or continuous basis, "surveillance projects" which is observation of the incidence in short term (early warning) or long term to observe trends over time and to make statistics on annual levels [2-4,8,9]. One methodological paper describes a minimal standard for primary care based surveillance networks and lists seven criteria recommendations for their structure and operation [2]. Good technical systems used to collect data may also be a challenge [13-15]. However, some countries use reimbursement data, Health Insurance Register, or patient registries/records to create national statistics [16-18]. We have found no scientific publications describing the method of obtaining continuous data from out-of-hours services in detail.

The data from the three month pilot period show that there are large differences in contact patterns and activities between out-of-hours districts in Norway. The data concerning day time contacts outside weekends obviously reflect different organisational models and access to ordinary general practice. It should therefore be considered that data only from periods outside ordinary working hours be used when Watchtower results are analysed.

## Conclusion

There is a need for comparative data from the out-of-hours services in Norway. The Watchtower project is established to give data on a large scale basis about contacts to casualty clinics in Norway, and can thus be useful for national statistics, research, and also system improvements. The data are also important for reflection on and feedback from activities in the casualty clinics themselves. The pilot study shows that the procedures for collecting data and the quality of the data obtained are satisfactory. The municipalities chosen are sufficiently representative for Norway as a whole. Data from this project may give possibilities for making international comparisons.

## Competing interests

The author(s) declare that they have no competing interests.

## Authors' contributions

SH planned the project, EHH and SH established the project including the procedures for data collection. EHH and SH planned the analyses and EHH performed them. EHH drafted the manuscript which then was rewritten by EHH and SH. Both authors approved the final manuscript.

## Pre-publication history

The pre-publication history for this paper can be accessed here:


